# Altered Sphingolipid Hydrolase Activities and Alpha-Synuclein Level in Late-Onset Schizophrenia

**DOI:** 10.3390/metabo14010030

**Published:** 2023-12-31

**Authors:** Tatiana Usenko, Anastasia Bezrukova, Katerina Basharova, Galina Baydakova, Elena Shagimardanova, Nataliya Blatt, Albert Rizvanov, Oleg Limankin, Maxim Novitskiy, Natalia Shnayder, Artem Izyumchenko, Mikhail Nikolaev, Anna Zabotina, Anna Lavrinova, Darya Kulabukhova, Regina Nasyrova, Ekaterina Palchikova, Natalia Zalutskaya, Irina Miliukhina, Yury Barbitoff, Oleg Glotov, Andrey Glotov, Anastasia Taraskina, Nikolai Neznanov, Ekaterina Zakharova, Sofya Pchelina

**Affiliations:** 1 Department of Molecular Genetic and Nanobiological Technologies Research Center, Pavlov First Saint-Petersburg State Medical University, 197022 Saint Petersburg, Russia; usenko_ts@pnpi.nrcki.ru (T.U.); bezrukova_ai@pnpi.nrcki.ru (A.B.); izyumchenko_ad@pnpi.nrcki.ru (A.I.); nikolaev_ma@pnpi.nrcki.ru (M.N.); zabotina_am@pnpi.nrcki.ru (A.Z.); kulabuhovadarya@gmail.com (D.K.); milyukhinaiv@yandex.ru (I.M.); taraskina_ae@pnpi.nrcki.ru (A.T.); pchelina_sn@pnpi.nrcki.ru (S.P.); 2 Petersburg Nuclear Physics Institute Named by B.P. Konstantinov of National Research Centre Kurchatov Institute, 188300 Gatchina, Russiagb2003@yandex.ru (G.B.); lavrinova_ao@pnpi.nrcki.ru (A.L.); 3 Research Center for Medical Genetics, 115478 Moscow, Russia; 4 Institute of Fundamental Medicine and Biology, Kazan Federal University, 420008 Kazan, Russia; rjuka@mail.ru (E.S.); nataliya.blatt@gmail.com (N.B.); rizvanov@gmail.com (A.R.); 5 Division of Medical and Biological Sciences, Tatarstan Academy of Sciences, 420111 Kazan, Russia; 6 Psychiatric Hospital No. 1 Named after P. P. Kashchenko, 195009 Saint Petersburg, Russia; limankin@mail.ru; 7 North-Western Medical University Named after P. I.I. Mechnikov of the Ministry of Health of the Russian Federation, 191015 Saint Petersburg, Russia; 8 Center for Personalized Psychiatry and Neurology of the N.N. V.M. Bekhtereva, 192019 Saint Petersburg, Russia; maximnovitsky93@gmail.com (M.N.); naschnaider2022@yandex.ru (N.S.); nreginaf77@gmail.com (R.N.); nezn@bekhterev.ru (N.N.); 9 V.M. Bekhterev National Medical Research Center Psychiatry and Neurology, 192019 Saint Petersburg, Russia; ofcoursekate@gmail.com (E.P.); nzalutskaya@yandex.ru (N.Z.); 10 Institute of the Human Brain of RAS, 197022 Saint Petersburg, Russia; 11 D.O. Ott Research Institute for Obstetrics, Gynecology, and Reproductology, 199034 Saint Petersburg, Russia; barbitoff@bk.ru (Y.B.); olglotov@mail.ru (O.G.); anglotov@mail.ru (A.G.); 12 Cerbalab Ltd., 197136 Saint Petersburg, Russia; 13 Bioinformatics Institute, 197342 Saint Petersburg, Russia; 14 Pediatric Research and Clinical Center of Infectious Diseases, 197022 Saint Petersburg, Russia; 15 School of Medicine, St. Petersburg State University, 199034 Saint Petersburg, Russia

**Keywords:** schizophrenia, Parkinson’s disease, hydrolase activity, sphingolipids, alpha-synuclein, lysosomal storage disorder genes

## Abstract

Recent data described that patients with lysosomal storage disorders (LSDs) may have clinical schizophrenia (SCZ) features. Disruption of lipid metabolism in SCZ pathogenesis was found. Clinical features of schizophrenia (SCZ) have been demonstrated in patients with several lysosomal storage disorders (LSDs). Taking into account the critical role of lysosomal function for neuronal cells’ lysosomal dysfunction could be proposed in SCZ pathogenesis. The current study analyzed lysosomal enzyme activities and the alpha-synuclein level in the blood of patients with late-onset SCZ. In total, 52 SCZ patients with late-onset SCZ, 180 sporadic Parkinson’s disease (sPD) patients, and 176 controls were recruited. The enzymatic activity of enzymes associated with mucopolysaccharidosis (alpha-L-Iduronidase (*IDUA*)), glycogenosis (acid alpha-glucosidase (GAA)) and sphingolipidosis (galactosylceramidase (*GALC*), glucocerebrosidase (GCase), alpha-galactosidase (GLA), acid sphingomyelinase (ASMase)) and concentration of lysosphingolipids (hexosylsphingosine (HexSph), globotriaosylsphingosine (LysoGb3), and lysosphingomyelin (LysoSM)) were measured using LC-MS/MS. The alpha-synuclein level was estimated in magnetically separated CD45+ blood cells using the enzyme-linked immunosorbent assay (ELISA). Additionally, NGS analysis of 11 LSDs genes was conducted in 21 early-onset SCZ patients and 23 controls using the gene panel PGRNseq-NDD. Decreased ASMase, increased GLA activities, and increased HexSpn, LysoGb3, and LysoSM concentrations along with an accumulation of the alpha-synuclein level were observed in late-onset SCZ patients in comparison to the controls (*p* < 0.05). Four rare deleterious variants among LSDs genes causing mucopolysaccharidosis type I (*IDUA* (rs532731688, rs74385837) and type III (*HGSNAT* (rs766835582)) and sphingolipidosis (metachromatic leukodystrophy (*ARSA* (rs201251634)) were identified in five patients from the group of early-onset SCZ patients but not in the controls. Our findings supported the role of sphingolipid metabolism in SCZ pathogenesis. Aberrant enzyme activities and compounds of sphingolipids associated with ceramide metabolism may lead to accumulation of alpha-synuclein and may be critical in SCZ pathogenesis.

## 1. Introduction

Schizophrenia (SCZ) is a mental disorder with a prevalence of 0.7–1% in the general population [1]. SCZ is attributed to hyperactive dopamine transmission that comprises an increase in dopamine synthesis capacity, higher synaptic dopamine levels, and augmented dopamine release [2]. The heritability of SCZ is estimated to be 70–90% [3]. Polymorphism heritability estimates suggest that less than 30% of genetic variations of SCZ are variants with minor allele frequency (MAF) of <1% in the population [4]. However, known genetic risk factors cannot explain the molecular mechanisms underlying the pathogenesis of SCZ. Recent advances from genome-wide association studies (GWAS) point to a broad polygenic network of brain-expressed genes contributing to SCZ pathogenesis [5,6,7]. 

The association of lysosomal storage disorders (LSDs) genes with a group of neurodegenerative disorders, namely synucleinopathies such as Parkinson’s disease (PD), dementia with Lewy bodies (DLB), and multiple system atrophy (MSA), is now widely discussed [8,9,10,11,12,13,14]. Previously, we and other research groups revealed alterations in lysosomal enzyme activities and sphingolipid concentrations in blood and brain tissues of patients with PD and also in DLB and MSA patients [15,16]. Transcriptome analysis of post-mortem brain tissues of SCZ patients demonstrated the dysregulation of genes involved in lysosomal function and cytoskeleton remodeling, suggesting the role of lysosomal dysfunction in SCZ pathogenesis [17]. Lipidomic analysis revealed alterations in lipid composition in SCZ patients compared to controls in the brain, biofluids (blood, cerebrospinal fluid (CSF), urea, plasma, and serum [18]. However, the assessment of lysosomal enzyme activities in the blood of SCZ patients has not been conducted. However, SCZ and PD do coexist rarely in clinical practice [2]. A recent study demonstrated that an increased genetic risk of PD may be associated with an increased risk of SCZ [19]. This association supports the intrinsic nature of the psychotic symptom of PD rather than medication or environmental effects [19]. Further studies are needed to support possible comorbidity in these diseases.

Given the above, the aim of the present study was to estimate the lysosomal hydrolases activities, lysosphingolipids concentrations, and alpha-synuclein level in blood cells of SCZ patients along with searching for rare pathogenic variants in LSDs genes using NGS analysis. 

## 2. Materials and Methods

All samples had been deposited at the Moscow Branch of the Biobank “All-Russian Collection of Biological Samples of Hereditary Diseases” (Research Centre for Medical Genetics, Moscow, Russia).

### 2.1. Samples 

In total, 52 late-onset SCZ patients, 180 sporadic PD patients with no family history of the disease (sPD), and 176 individuals without neurological disorders were included in the study of lysosomal hydrolase activities. Patients with SCZ were diagnosed at two clinic centers of Saint Petersburg, St. Petersburg Psychiatric Hospital No. 1, named after P. P. Kashchenko, and V.M. Bekhterev National Medical Research Center for Psychiatry and Neurology. Standard neurologic clinical examination was performed for all participants, and the diagnosis of SCZ was based on the International Classification of Diseases, 10th revision (ICD-10). 

Additionally, 23 male patients with early-onset SCZ and the first episode of SCZ (age at exam: 30.95 ± 8.82 y.o.) and also 21 neurological healthy male individuals (age at exam: 35.86 ± 9.02 y.o.) without bipolar disorder or SCZ (Controls_NGS) were enrolled into the analysis with targeted NGS to search for rare variants among LSDs genes. Unrelated healthy individuals with no family history of the neurological disorders were recruited as controls. A group of sPD patients was generated in three neurological clinic centers of St. Petersburg: Pavlov First Saint-Petersburg State Medical University, Institute of experimental university, Institute of the Human Brain of RAS. The demographic characteristics of the studied groups are presented in Table 1. 

### 2.2. Assessment of Enzyme Activities and Lysosphingolipid Concentrations in Blood 

From each of the study participants, fresh venous blood samples were collected in EDTA tubes. Dry blood spots (DBS) cards were prepared by pipetting 40 µL of whole blood on each spot. DBS were allowed to dry in open air at room temperature for 2 h and then were stored at −20 °C until extraction. Enzyme activities of alpha-L-Iduronidase (IDUA, EC 3.2.1.76, deficient in Mucopolysaccharidosis Type I) and also galactosylceramidase (GALC, EC 3.2.1.46, deficient in Krabbe disease), glucocerebrosidase (GCase, EC 3.2.1.45, deficient in Gaucher disease), alpha-galactosidase A (GLA, EC 3.2.1.22 deficient in Fabry disease), acid sphingomyelinase (ASMase, EC 3.1.4.12, deficient in Niemann-Pick disease types A and B), and acid alpha-glucosidase (GAA, EC 3.2.1.20, deficient in Pompe disease), and concentrations of lysosphingolipids (hexosylsphingosine (HexSph) (glucosylsphingosine (GlcSph) + galactosylsphingosine (GalSph)), globotriaosylsphingosine (LysoGb3), and lysosphingomyelin (LysoSM)) were estimated by liquid chromatography tandem-mass spectrometry (LC-MS/MS) in dry blood spots (DBS) as described earlier [20].

### 2.3. Detection of Alpha-Synuclein Level in CD45+ Blood Cells 

CD45+-cells were isolated from 8 mL fresh peripheral blood by density gradient centrifugation (Ficoll-Paque PLUS, GE Healthcare, Chicago, IL, USA) followed by magnetic sorting using CD45+ MicroBeads and miniMACS columns type MS (Miltenyi Biotec, Bergisch Gladbach, Germany) according to the manufacturer’s instructions. The cell suspension was aliquoted and frozen at −70 °C. Alpha-synuclein level in CD45+-cells was determined by ELISA using Human alpha-synuclein ELISA kit (Thermo Fisher Scientific, Waltham, MA, USA). The cells were lysed with Total Protein Extraction Kit (Chemicon (Millipore, Burlington, MA, USA). The total protein concentration was measured with Pierce BSA Protein Assay kit (ThermoScientific, Waltham, MA, USA). Samples adjusted to 6 µg of total protein were used in experiments. Each sample was evaluated in triplicate. Optical density was measured using microplate spectrophotometer xMark (Bio-Rad, Hercules, CA, USA). Homogeneous cell fraction of CD45+ cells was used because the red blood cells may distort the results because they contain more than 99% of alpha-synuclein in the total blood fraction [21].

### 2.4. Next Generation Sequencing and Variant Calling Analysis of LSDs Genes

After sample collection, blood DNA samples were isolated from each individual included in the current study. An amount of 100 ng of DNA was used to generate sequencing libraries using the KAPA HyperPlus kit (Roche, Basel, Switzerland) using enzymatic fragmentation according to manufacturer’s instructions. The size of library fragment was evaluated using Agilent 2100 Bioanalyzer (Agilent technologies, Santa Clara, CA, USA). The NimbleGen SeqCap EZ Choice kit (Roche, Basel, Switzerland) was used for target enrichment. The gene panel PGRNseq-NDD was specifically designed for targeted sequencing of genes implicated in inflammation, immunoreactivity, neurodegeneration, metabolism, and detoxification of drugs, xenobiotics, and endogenous substances and was described in our previous study [22]. Sequencing was performed using Illumina MiSeq platform with generation sequence reads, producing paired-end reads spanning 250 bases on average. Sequencing of controls 1 was carried out on the Illumina Hiseq 1500 NGS platform with libraries prepared using Illumina exome kits. Quality control for each sample was performed by FastQC (v0.11.9). In this step, clean reads were obtained by pre-processing the raw reads with Trimmomatic (v0.36). All downstream analyses, such as alignment and variant calling, were based on the high-quality clean data.

To map paired-end reads passing the pre-processing onto the human reference genome build GRCh37 (Gencode, https://www.gencodegenes.org, accessed on 10 January 2023), the Burrows–Wheeler Aligner (BWA) (v0.7.17) was used [23]. The identification of single nucleotide variants (SNVs) and small insertions/deletions (indels) in individual BAM files was performed using the Genome Analysis Toolkit (GATK) (v 4.2.6.1) [24]. SNVs and indels were annotated using the ANNOVAR tool [25]. The pathogenicity of variants was tested using prediction algorithms such as SIFT, PolyPhen-2-HDIV, Mutation taster, Mutation assessor, and LRT and scores to measure predicted pathogenicity, as was described earlier by Ganesh and colleagues [26]. Deleterious variants were selected and their prioritization was carried out as follows: Variant predicted as deleterious by all five prediction algorithms or by one or more of the five prediction algorithms. Identified variants were filtered for false positives by removing variants not passing all filters as residing in intronic and intergenic regions, and causing a synonymous, non-frameshift change. Finally, variants were removed from analysis if their MAF was more than 1% in Exome Aggregation Consortium—ALL (ExAC-ALL) and non-Finnish European ExAC (ExAC-NFE) [27], read depth ≤ 80, and variant quality value ≤ 20. All determined variants were completely absent from our controls. The block diagram of pipeline is presented on supplementary Figure S1. The variants of genes associated with inherited metabolic disorders were validated by Sanger sequencing. Sequences of primers are available in Supplementary Table S1. 

### 2.5. Statistical Analysis

Conformity of findings to normal distribution was tested using the Shapiro–Wilk test. Activity of each enzyme was compared between studied groups using the nonparametric Mann–Whitney u-test. Significance with Bonferroni correction for multiple comparisons was established at *p* < 0.05. For odds ratio, logistic regression analysis was used, in which SCZ status was the outcome and enzymatic activities, lysosphingolipid concentrations, and alpha-synuclein level were the predictors, adjusted for age and sex, and disease duration. Next, we divided SCZ patients into four groups based on quartiles of enzymatic activities, lysosphingolipid concentrations, and alpha-synuclein level measured in the control group, and ANOVA was performed to examine the association between enzymatic activities and AAO of disease, as was performed earlier [16,28]. Statistical analysis was performed using R software (version 4.1.2). Clinical data and experimental data are expressed as the mean ± SD. 

## 3. Results

### 3.1. Lysosomal Enzymatic Activities, Lysosphingolipid Concentrations, and Alpha-Synuclein Level in Patients with Late-Onset SCZ

In our study, the enzymatic activities of GCase, ASMase, GLA, GALC, GAA, and IDUA lysosomal enzymes were estimated by means of multiplex assay based on LC-MS/MS, as described earlier [20].

The estimated enzymatic activities of lysosomal enzymes (GCase, ASMase, GLA, GALC, GAA, IDUA) in the blood of patients with late-onset SCZ, sPD patients, and controls are presented in Table 2 and Supplementary Figure S2. ASMase activity was decreased in patients with late-onset SCZ in comparison to the sPD patients and controls (*p* < 0.00001). GAA activity was decreased in the blood of patients with late-onset SCZ compared to sPD (*p* = 0.019). GALC activity was increased in the blood of patients with late-onset SCZ and sPD compared to controls (*p* < 0.05). Unexpectedly, GLA activity was increased in the late-onset SCZ patients compared to sPD patients and controls (*p* < 0.00001). Also, decreased IDUA activity was found in the late-onset SCZ patients compared to the sPD patients and controls. However, the difference did not reach statistical significance (*p* = 0.085, *p* = 0.074, respectively) (Table 2, Supplementary Figure S2). No significant differences in GCase and IDUA activities between all studied groups were found (*p* > 0.05). 

Lysosphingolipid concentrations (HexSph, LysoGb3, LysoSM) in the blood were estimated by means of multiplex assay based on LC-MS/MS, as described earlier [29,30]. The concentrations of all studied lysosphingolipids were increased in patients with late-onset SCZ compared to sPD and controls (*p* < 0.00001) (Table 2, Supplementary Figure S3). As was reported earlier, sPD patients were characterized by decreased LysoSM concentration compared to controls (*p* = 0.00021). HexSph and LysoGb3 did not differ between sPD patients and controls (*p* > 0.05) [16] (Table 2, Supplementary Figure S3).

**Figure 1:**
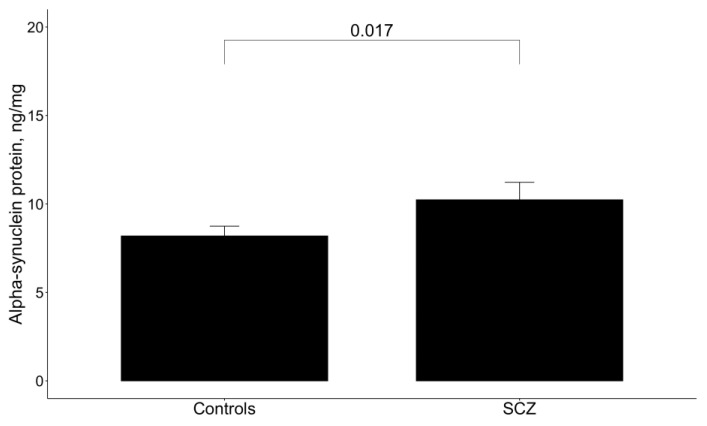
Alpha-synuclein level in CD45+ blood cells of late-onset SCZ patients and controls.

The alpha-synuclein level in CD45+ blood cells was measured using ELISA in the studied groups. As was reported earlier, the alpha-synuclein level was increased in sPD patients compared to controls (*p* = 0.024) [31]. Interestingly, the patients with late-onset SCZ (10.24 ± 0.99) were also characterized by elevated alpha-synuclein levels compared to controls (8.19 ± 0.55) (*p* = 0.017) (Figure 1).

### 3.2. Correlation Analysis of Lysosomal Enzyme Activities, Lysosphingolipid Concentrations, and Alpha-Synuclein Level in Patients with Late-Onset SCZ 

Correlation analysis was performed in order to assess whether there was an association among enzymes activities, lysosphingolipid concentrations, and alpha-synuclein level in SCZ patients (Supplementary Figure S4). At first, correlation analysis was conducted. Positive correlations between all enzymes were found in the group of SCZ patients (Supplementary Figure S4). For lysosphingolipid concentrations, a negative correlation between HexSph concentration and LysoSM concentration (*p* = 0.022, r = 0.35) was found in the SCZ patients. Although, HexSph concentration was positively correlated with GALC activity (*p* = 0.01, r = 0.35) in the SCZ patients. Despite an elevation in the alpha-synuclein level in the SCZ patients, no correlations with lysosphingolipid concentrations and enzyme activities were found (*p* > 0.05) (Supplementary Figure S4).

Because correlations between activities of lysosomal enzymes were found, multivariable logistic regression analysis was performed to identify the contribution of enzymatic activities to SCZ status in patients, adjusted for age and sex (Table 3). Higher ASMase activity was associated with lower odds of SCZ status (OR = 0.349, 95%CI: 0.204–0.599; *p* = 0.0001). Surprisingly, increased GLA activity was found to be associated with an increased SCZ risk in SCZ patients (OR = 1.162; 95%CI: 1.818–1.809; *p* = 0.0004) (Table 3). There was no association among GCase, GALC, GAA, and SCZ status. 

The same analysis was conducted for lysosphingolipid concentrations and alpha-synuclein levels. A strong association between SCZ status and increased concentrations of ASMase and GLA substrates, LysoSM, and LysoGb3, respectively (OR = 1.059, 95%CI: 1.037–1.081; *p* = 1.95 × 10^−7^; OR = 1.290, 95%CI: 1.092–1.523; *p* = 0.0032, respectively) and also of substrates GCase and HexSph (OR = 1.072, 95%CI: 1.047–1.098; *p* = 4.53 × 10^−8^) were found. A higher alpha-synuclein level was associated with higher odds of SCZ status (OR = 1.017, 95%CI: 1.006–1.028; *p* = 0.0027) (Table 3).

Additionally, the association of the studied parameters with the value of the PANSS scale, adjusted for sex and age, was assessed using regression analysis. However, the association was not identified (*p* > 0.05). Data are not provided. Correlation analysis also did not reveal any difference in PANSS values and all studied parameters (Figure S4). Multiple regression analysis did not show any association between the values of the MoCA scale and all studied parameters as well. However, correlation analysis showed a negative correlation between GLA, GAA activities, and HexSph concentration and MoCA value (*p* = 0.014, r = −0.34, *p* = 0.011, r = −0.35, *p* = 0.032, r = 0.30, respectively) and a positive correlation between LysoSM concentration and the MoCA value (*p* = 0.007, r = 0.37) (Figure S4).

### 3.3. Enzyme Activities, Lysosphingolipid Concentrations, and Alpha-Synuclein Level Are Associated with the Age at Onset of SCZ

We divided SCZ patients to four groups, based on the enzymatic activities, lysosphingolipid concentrations, and the alpha-synuclein level quartiles in controls (Supplementary Table S2). Enzymatic activities of IDUA, GCase, GAA, ASMase, and GLA were not associated with age at onset (AAO). Surprisingly, we revealed that increased GALC activity was associated with an earlier AAO, with significant differences of more than 11 years in patients with late-onset SCZ (*p* = 0.029) (Supplementary Table S2). No associations among lysosphingolipid concentrations, alpha-synuclein level, and AAO of patients with SCZ were found (*p* > 0.05) (Supplementary Table S2). 

### 3.4. Selection of Rare Deleterious Variants in Patients with Early-Onset SCZ 

Additionally, target sequencing analysis was conducted for 23 early-onset SCZ patients and 21 neurological healthy individuals (Controls_NGS). 

Four variants of eleven LSDs genes were selected in group 1 using the pipelines described in Section 2, namely *IDUA* (rs532731688, rs74385837), *ARSA* (rs201251634), and *HGSNAT* (rs766835582) (Supplementary Table S3). 

Variants of *ARSA* (rs201251634) and *HGSNAT* (rs766835582) were selected because they were determined as deleterious by all five predictors (SIFT, PolyPhen-2 HDIV, Mutation taster, Mutation assessor, and LRT); *IDUA* (rs74385837) was determined as deleterious by three predictors (PolyPhen-2 HDIV, Mutation taster, Mutation assessor, LRT), and *IDUA* (rs532731688). All variants were detected in a heterozygous state. Mutations in *IDUA* and *HGSNAT* cause mucopolysaccharidosis I and III types, respectively; mutations in *ARSA* cause metachromatic leukodystrophy (MLD) that belongs to the sphingolipidosis group. All selected genes are expressed in brain tissues and in others (https://www.proteinatlas.org, accessed on 1 November 2023). No variants of all LSDs genes [11] were found in Controls_NGS as all selected variants were located in the genes (*IDUA*, *HGSNAT* and *ARSA*) linked with mucopolysaccharidosis and sphingolipidosis, respectively.

## 4. Discussion

**Figure 2:**
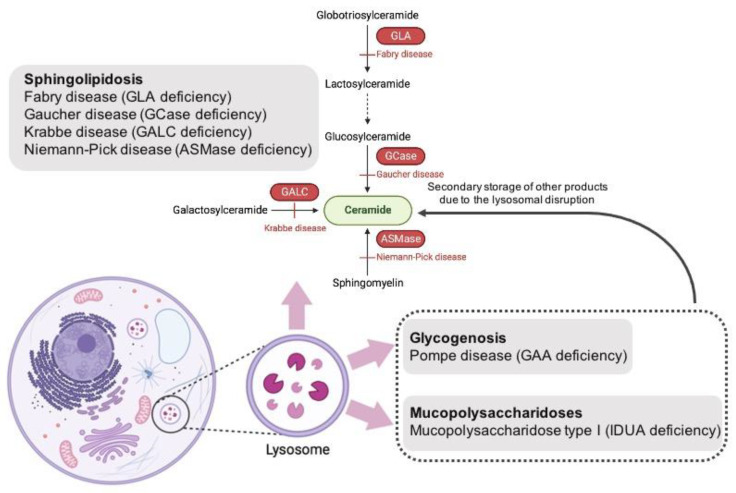
Hydrolase enzymes included in this study participate in ceramide metabolism in the lysosome as indicated by names in red.

The results of our study highlight the role of lysosomal dysfunction in SCZ pathogenesis. We first focused on an estimation of enzymatic activities of lysosomal hydrolases encoded by genes causing the mucopolysaccharidosis (IDUA) and sphingolipidoses (GCase, ASMase, GALC, GLA) and additional glycogen storage disorders (glycogenosis) (GAA) in patients with late-onset SCZ. Among all studied enzymes, the impaired activities of ASMase, GLA, GALC, and GAA and the increased concentration of lysosphingolipids (HexSph, LysoGb3, LysoSM) were shown in late-onset SCZ patients compared to controls. It is interesting to note that all enzymes and lysosphingolipids are involved in ceramide metabolism (Figure 2). 

It is well known that ceramides are enriched in neural tissues and are important for brain functioning [32]. The disturbances in sphingolipid metabolism in neurodegeneration is now widely discussed [33]. Altered enzymatic activities of hydrolyzes and sphingolipid compounds taking part in ceramide metabolism were demonstrated in the blood of sPD patients as well as in patients with other synucleinopathies (DLB, MSA) by us and others in previous studies [15,16]. In a postmortem study, Moors and coauthors demonstrated a decrease in GCase activity in the substantia nigra of sPD and DLB patients [15]. Several studies demonstrated decreased GCase activity in the blood of sPD patients. 

It is interesting to note that synucleinopathies could in some cases be presumed as a comorbid condition in patients with SCZ [2,34,35,36]. Earlier, the postmortem studies demonstrated an increase in ceramides as hydroxyceramides, phytoceramides, hexosylceramides, lactosylceramides, and ceramide phosphoethanolamines and others in white matter and on the frontal cortex of SCZ patients [37,38]. Recently, in blood plasma, the increase in Cer (d18: 1/16: 0), Cer (d18: 1/18: 0), and Cer (d18: 1/24: 1) was demonstrated in SCZ patients compared to controls [32]. Abnormalities of ceramides were associated with cognitive impairments in SCZ patients [39]. So, ceramide is involved in different physiological and pathological cellular processes [40] and may be a part of SCZ pathogenesis, in particular, due to the impairment of myelin formation and oligodendrocyte dysfunction and neuroinflammation [41,42]. 

In the present study, we first revealed decreased ASMase and increased GLA activities which were accompanied by the accumulation of appropriate substrates (LysoSM, LysoGb3, respectively) in the blood of late-onset SCZ patients compared to controls and sPD patients. 

ASMase is a lysosomal hydrolase responsible for the breakdown of sphingomyelin into ceramide and phosphocholine in the lysosome and on the plasma membrane [43,44]. In our previous study, we demonstrated a decrease in ASMse activity in the blood of MSA patients [16]. Interestingly, a recent review discussed the abnormalities of the ASMase–ceramide signaling pathway in SCZ patients [45]. This review discusses that the aberrations in the ASMase/ceramide system, especially ASMase activity and the levels of ceramide, may alter the cerebral microdomain structure and neuronal metabolism, leading to neurotransmitter dysfunction as dopamine neurotransmission and neuroinflammation [45]. Sphingomyelin is a critical component of the myelin sheath. Previously, an aberrant level of sphingomyelin and its metabolite, such as ceramide, was associated with cognitive impairments in SCZ patients [39]. And oppositely, several LSGs are characterized by psychotic behavior. Thus, Niemann-Pick disease (NPD) types A and B is an LSD caused by ASMase deficiency, which catalyzes the hydrolysis of sphingomyelin (SM) to ceramide and phosphocholine. Niemann-Pick disease type C (NPC) is a rare progressive genetic disorder characterized by an inability of the body to transport cholesterol and lipids inside of cells. NPC may be characterized by psychotic behavior, such as somatic hallucinations leading to the diagnosis of schizophrenia-like psychosis. However, earlier research has described a broad range of neurological manifestations also for NPD including severe ataxia, cognitive deficits, and psychiatric disorders [46]. Zhuo and coauthors suggested that normalization of the aberrant ASMase/ceramide system or ceramide reduction using approved functional inhibitors of ASMase, such as fluvoxamine and rosuvastatin, may improve clinical outcomes of SCZ patients [45]. 

At the same time, we first found increased GLA activity in SCZ patients compared to sPD patients and controls, and also, elevated GLA activity and its substrate (LysoGb3) concentration were associated with an increased risk of SCZ. GLA deficiency due to mutations in the *GLA* gene causes Fabry disease and leads to an accumulation of neutral glycosphingolipids in lysosomes within various tissues including the nervous system [47]. 

The reason for increased GLA activity and decreased ASMase activity and the accumulation of LysoGb3 and LysoSM in SCZ patients is currently unclear. GLA and ASMase and their substrates LysoGb3 and LysoSM are directly involved in ceramide metabolism (Figure 2). A decrease in ASMase activity and an increase in the activity of GLA, along with changes in the concentrations of their substrates (LysoSM, LysoGb3, respectively) may reflect a disturbance in ceramide metabolism in patients with SCZ. According to our results and the hypothesis of Zhuo and colleagues, it is likely that not only may the ASMase-ceramide signaling pathway be involved in SCZ, but aberrant GLA-ceramide signaling pathways may also be associated with SCZ. Further studies are needed to test these results. 

Additionally, we first revealed elevated GALC activity in SCZ patients compared to controls. An increase in GALC activity was associated with decreased AAO in SCZ patients. Galactosylceramide (GalCer) is synthesized when galactose is added to the 1-hydroxyl moiety of ceramide. GALC catalyzes the hydrolysis of GalCer and galactosylsphingosine. A deficiency of activity of lysosomal enzyme GALC in patients with the homozygous GALC mutation leading to Krabbe disease results in the rapid accumulation of galactosylsphingosine (or psychosine), a neurotoxic sphingolipid to neurons and myelinating cells [48]. GALC as ASMase and GLA is involved in sphingolipid and ceramide metabolisms (Figure 2). An increase in GALC activity in SCZ patients may correlate with an increase in myelin level [49]. Recently, it was shown that GALC interacts with sphingolipid activator proteins (SapA) with the formation of the heterotetramer complex mediated lipid catabolism in the lysosome [50]. SapA lipoprotein can solubilize phospholipids, sphingolipids, and cholesterol into discrete, monodisperse particles [51]. Alteration of GALC activity may influence lipid metabolism, in particular, phospholipid metabolism [52]. 

As well, here, decreased GAA activity in the blood of patients with SCZ compared to patients with sPD was found. A deficiency of GAA causes an autosomal recessive disease, Pompe disease, which belongs to the group of glycogen storage disorders. GAA catalyzes the hydrolysis of glycogen. GAA is expressed in skeletal muscle, heart, kidneys, and CNS [53]. Glucose derived from glycogen hydrolyzed by GAA enters the glycolytic pathway to generate pyruvate and subsequently acetyl-CoA, the fundamental building block of cholesterol and fatty acids. Fatty acids are needed for de novo synthesis of ceramide, cerebrosides, sphingolipids, and glycosphingolipids [53]. Pompe disease is characterized by myelin abnormalities that may occur due to impaired ceramide metabolism [54,55]. So, GAA is important for sphingolipid metabolism, and as a consequence, for ceramide metabolism. 

We did not reveal an alteration in GCase activity in SCZ patients, although the activity of this hydrolase positively correlated with the activity of other enzymes included in the study of SCZ patients (Supplementary Figure S4). GCase hydrolyses glucosylceramide to ceramide and glucose. Gaucher disease (GD) is a rare LSD caused by mutations in the *GBA1* gene, which results in deficient lysosomal enzyme glucocerebrosidase (GCase) activity. Previously, decreased GCase activity was demonstrated in blood and brain samples of PD and DLB patients compared to controls [15,56], and at the same time, we and other researchers did not reveal a decrease in GCase activity in blood samples of PD patients [20,57]. Nevertheless, in the current study, increased HexSph concentration, which is a substrate of GCase, was found in late-onset SCZ patients compared to sPD patients and controls which supports the data about the role of ceramide metabolism in SCZ pathogenesis (Figure 2) [58]. Our data support the role of ceramide metabolism in SCZ pathogenesis. Eventually, the described alterations were more pronounced in SCZ than in PD.

In the second part, we assessed the CD45+ alpha-synuclein level in SCZ patients and controls. Elevated alpha-synuclein levels were found in CD45+ blood cells of late-onset SCZ patients compared to controls. Alpha-synuclein protein encoded by the *SNCA* gene plays a pivotal role in PD pathogenesis and is one of the most abundant proteins in the nervous system and regulates the key stages of dopamine homeostasis [59,60]. In turn, dysregulation of dopamine homeostasis is implicated in neurodegenerative diseases such as PD, drug addiction, and neuropsychiatric disorders such as SCZ [61]. Earlier, we and others found alpha-synuclein accumulation in blood mononuclear cells in patients with PD compared to controls [31,62,63,64]. The role of alpha-synuclein in SCZ pathogenesis remains controversial [65,66,67,68]. In one study, the expression level of alpha-synuclein did not differ between controls and SCZ patients in peripheral blood lymphocytes [65]. In another study, serum alpha-synuclein levels were reduced in patients with SCZ compared to controls [68]. Interesting, one case study found that ten years after SCZ onset, the disease progressed to mild parkinsonism in patients with a duplication of the *SNCA* gene [69]. *SNCA* dosage is responsible for parkinsonism and leads to an increased level of alpha-synuclein protein [70,71,72]. However, studies evaluating alpha-synuclein included SCZ patients with an AAO of 30 years, which refers to early-onset SCZ. Similar studies evaluating the level of alpha-synuclein in a group of late-onset SCZ patients have not been previously conducted. 

Taken together, the alteration in the activity of enzymes (ASMase, GLA, GALC) and the concentration of lysisiphingolipids (HexSph, LysoGb3, LysoSM) involved in sphingolipid metabolism may affect the degradation of the alpha-synuclein protein, as a consequence of its accumulation in the cell [58,73]. Moreover, the direct influence of lysosphingolipids on alpha-synuclein aggregation was shown in in vitro studies [74]. However, it is important to mention that GlcCer was shown to induce mild aggregation of monomeric alpha-synuclein, but it primarily acts on alpha-synuclein oligomeric species and directly converts them into toxic oligomers with more compact conformation [75].

Additionally, we conducted a search for rare pathogenic variants in the LSD genes of a group of patients with early-onset SCZ. All rare variants of LSDs genes in the present study were found in a heterozygous state in SCZ patients and were not found in controls. Despite the fact that most LSDs are autosomal-recessive disorders, even one pathogenic variant in LSDs genes could highly increase the risk, for example, of neurodegenerative disorders. Thus, mutations in the *GBA1* gene causing GD as well as in the *SMPD1* gene causing Nieman-Pick A/B (NPC A/B) can increase the risk of PD by 5-10 times [12,76,77,78,79]. Moreover, the cumulative impact of the mutations in LSDs genes on PD was proposed in burden analyses [11,14]. Our results supported the role of the *ARSA* gene in SCZ risk, as shown in the study of Trakadis and coauthors [80]. The *ARSA* gene (mapped at 22q13.33) encodes arylsulfatase. Mutations in the *ARSA* gene cause the autosomal-recessive disorder metachromatic leukodystrophy (MLD) (OMIM #250100) due to a deficiency of arylsulfatase A activity. A partial or complete deficiency of arylsulfatase A activity results in an accumulation of sulfatides in the central and peripheral nervous systems that leads to the demyelination of axons and peripheral nerves [81]. Interestingly, patients with MLD are often diagnosed with SCZ [82]. It is worth noting that in the above-mentioned study of Trakadis and coauthors, among the thirteen IEM genes associated with SCZ, five of them belong to LSDs genes, and four of them (*HEXA*, *NCP1*, *NCP2*, *ARSA*) are causative of sphingolipidosis [80]. We first described the rare deleterious variants of the *HGSNAT* and *IDUA* genes in SCZ patients. Mutations in the *HGSNAT* gene cause mucopolysaccharidoses III type or Sanfilippo syndrome type C due to a deficiency of acetyl-CoA a-glucosaminide N-acetyltransferase (EC 2.3.1.78) that catalyzes the degradation of heparan N-sulfatase (sulfamidase). Patients with Sanfilippo syndrome have psychiatric manifestations. Mutations in the *IDUA* gene cause mucopolysaccharidosis I (MPS I) due to a deficiency of a-L-iduronidase (IDUA) that leads to defective catabolism of the glycosaminoglycans such as heparan and dermatan sulphate [83,84]. MPSI manifests as varying degrees of intellectual impairment, neuropathology, and neurological manifestations [85]. Here, the tendency for decreased IDUA activity in the blood of late-onset SCZ patients compared to sPD patients and controls was observed (Table 2, Supplementary Figure S2). Thus, our results are in agreement with the association of LSD genes with SCZ. Mutations in the *ARSA*, *HGSNAT,* and *IDUA* genes were also found in patients with PD which co-occurs with SCZ [11,86]. 

The limitations of the current study are the sample size of the studied groups of patients with SCZ and the controls for NGS analysis. Larger cohorts are needed to robustly identify genes that putatively contribute to SCZ risk in the Russian population and in others. The next limitation is the fact that NGS sequencing and an estimation of lysosomal enzymes activities and lysosphingolipid concentrations were conducted in different groups of SCZ patients. The groups were different in terms of sex and age. Further studies on lysosomal enzyme activities in a group of early-onset SCZ patients are needed. Another limitation is the lack of information on treatment and body mass index for the late-onset SCZ patients.

## 5. Conclusions

The results of the current study suggest an association between metabolic pathways and LSDs and SCZ. Pronounced aberrant activities of ASMase and GLA and accumulation of their substrates (LysoSM, LysoGb3) and also HexSph may play a role in late-onset SCZ pathogenesis and support the interruption of ceramide metabolism in SCZ that, in turn, may be associated with the identified accumulation of alpha-synuclein protein. Rare variants in genes causing sphingolipidosis (*ARSA* (rs201251634) and *HGSNAT* (rs766835582)) and mucopolysaccharidosis (*IDUA* (rs532731688, rs74385837) were revealed in SCZ patients but not in controls. Further studies are needed to confirm our results.

## Figures and Tables

**Table 1:** Demographic and clinical characteristics of the compared groups.

Groups	Age at Exam, Mean ± SD, Years	Age at Onset, Mean ± SD, Years	Sex (Male–Female)	Positive and Negative Syndrome Scale (PANSS)	Montreal Cognitive Assessment (MoCA)
Patients with SCZ with late-onset (N = 52)	61.0 ± 11.1	51.1 ± 11.5	20:32	75.9 ± 15.8	25.9 ± 2.39
Patients with sPD (N = 180)	63.5 ± 9.2	57.6 ± 10.2	75:105	-	-
Controls (N = 176)	62.4 ± 8.9	-	70:106	-	-
Patients with SCZ with early-onset_NGS (N = 23)	31.4 ± 8.75	NA	23:0	NA	NA
Controls_NGS (N = 21)	35.5 ± 9.06	-	21:0	-	-

NA—not applicable.

**Table 2:** Lysosomal enzyme activities and lysosphingolipid concentrations in blood cells of studied groups.

Groups	Estimated Parameters, Mean ± SE								
	Enzyme Activity in the Whole Blood, Mmol/L/h						Substrate Concentration in the Whole Blood, ng/mL		
	ASMase	GCase	GLA	GALC	IDUA	GAA	LysoSM	HexSph	LysoGb3
Patients with late-onset SCZ (N = 52)	2.79 ± 0.13 *p* = 3.2 × 10^−8^ * p = 1.8 × 10^−11^ **	6.78 ± 0.32	6.63 ± 0.45 *p* = 2.2 × 10^−6^ * p = 7.9 × 10^−5^ **	2.49 ± 0.13 *p* = 0.0054 *	6.71 ± 0.34	7.21 ± 0.45 *p* = 0.019 **	6.76 ± 0.58 *p* = 1.3 × 10^−7^ * p = 1.1 × 10^−12^ **	6.03 ± 0.36 *p* = 1.6 × 10^−9^ * p = 5.8 × 10^−11^ **	1.24 ± 0.06 *p* = 1.4 × 10^−10^ * p = 1.4 × 10^−8^ **
sPD (N = 180)	4.94 ± 0.17	7.65 ± 0.30	45.01 ± 0.26	2.50 ± 0.12 *p* = 0.011 *	7.53 ± 0.24	8.63 ± 0.33	3.53 ± 0.09 *p* = 0.00021 *	3.49 ± 0.19	0.86 ± 0.04
Controls (N = 176)	44.71 ± 0.18	7.42 ± 0.34	4.66 ± 0.18	2.15 ± 0.09	7.94 ± 0.28	8.30 ± 0.30	4.11 ± 0.12	3.59 ± 0.19	0.82 ± 0.03

*—compared to controls. **—compared to sPD.

**Table 3:** The association among lysosomal enzymes activities, lysosphingolipid concentrations, alpha-synuclein level, and SCZ status.

Group	Parameters	Odds Ratio	95% CI	*p*-Value
	Enzymatic activities			
Patients with SCZ with late- onset	GCase	0.994	0.971–1.018	0.6621
	GLA	1.051	1.025–1.077	0.0001
	ASMase	0.925	0.889–0.963	0.0002
	GALC	1.016	0.958–1.078	0.5786
	IDUA	0.998	0.970–1.027	0.9124
	GAA	1.004	0.980–1.0295	0.7201
	Age	1.000	0.993–1.007	0.8792
	Sex	0.999	0.871–1.145	0.9887
	Substrate concentrations			
	HexSph	1.072	1.047–1.098	4.53 × 10^−8^
	LysoGb3	1.290	1.092–1.523	0.0032
	LysoSM	1.059	1.037–1.081	1.95 × 10^−7^
	Age	0.997	0.991–1.004	0.4540
	Sex	0.976	0.862–1.106	0.7071
	Alpha-synuclein level			
	Alpha-synuclein	1.017	1.006–1.028	0.0027
	Age	1.093	0.989–1.004	0.4657
	Sex	0.997	0.944–1.264	0.2344

## Data Availability

The data discussed in this publication have been deposited at ArrayExpress database at EMBL-EBI https://www.ebi.ac.uk/biostudies/ (accessed on 13 December 2023) under accession number S-BSST1274 https://www.ebi.ac.uk/biostudies/studies/S-BSST1274 (accessed on 13 December 2023) for array design “S-BSST1274”.

## Supplementary Materials

The following supporting information can be downloaded at: https://www.mdpi.com/article/10.3390/metabo14010030/s1, Table S1: Sequences of primers for validation analysis by Sanger sequencing; Table S2: Activity of lysosomal enzymes, substrate concentrations, alpha-synuclein level AAO of SCZ; Table S3: List of selected rare deleterious variants of LSDs genes in SCZ patients, Figure S1: Block diagram of pipeline with numbers indicating the reduction in the total number of variants in each prioritization step; Figure S2: Activity of lysosomal enzyme in blood of studied groups. A. ASMase, B. GLA, C. GALC, D. GAA, E. IDUA, Figure S3: Substrate concentrations in blood and alpha-synuclein level in CD45+ blood cells of studied groups: A. HexSph, B. LysoGb3, C. LysoSM, Figure S4: Correlation matrix among enzymes activities, substrate concentrations, and alpha-synuclein level in patients with late-onset SCZ. The violet and green dots correspond to negative and positive correlations, correspondently. Small dots with light colors represent lower correlations. Larger dots with darker colors correspond to higher correlations. White square—no statistically significant association, *p* > 0.05).
