# Supplementary material for: Altered Sphingolipid Hydrolase Activities and Alpha-Synuclein Level in Late-Onset Schizophrenia
Source: Metabolites. 2023 Dec 31;14(1):30. doi: 10.3390/metabo14010030 (PMC10819534; doi:10.3390/metabo14010030)
Supplement: Supplementary file 1 [file metabolites-14-00030-s001.zip › metabolites-2778043-supplementary.pdf]

metabolites

MDPI

Supplementary Information

Table S1. Sequences of primers for validation analysis by Sanger sequencing.

Genes	Primers 3′-5′	Length, bp	PCR product size, bp
ARSA_rs201251634_FOR	AGAACCTGACCTGCTTCCC	19	302
ARSA_rs201251634_REV	AACTGAGGGTAGTGGGTGTG	20	
HGSNAT_rs766835582_FOR	GCTGAGCCCATGTCTCTTGA	20	399
HGSNAT_rs766835582_REV	ACTTGAAGCCAGGAGTGAGG	20	
IDUA_ rs74385837_FOR	TGAACTACTACGATGCCTGCT	21	323
IDUA_rs74385837_REV	AGGATGGAGATGGAGCTGC	19	
IDUA_ rsrs532731688_FOR	GATGAGGAGCAGCTCTGGG	19	343
IDUA_rsrs532731688_REV	GAGCCCGTTGTCCAGGTAG	19	

Table S2. Activity of lysosomal enzymes, substrate concentrations, alpha-synuclein level, and AAO of SCZ.

Groups	Lysosomal enzymes								
	**GCase**			**GLA**			**ASMase**		
Patients with late-onset SCZ	<4.57	53.5±14.0	0.314	<2.98	49.3±11.8	0.441	<2.75	52.8±12.8	0.286
	4.57-6.29	53.9±13.5		2.98-4.20	56.5±16.3		2.75-4.15	49.8±9.0	
	6.29-9.13	49.6±9.2		4.20-5.64	54.0±12.3		4.15-6.28	43.8±5.2	
	>9.13	40.0±0		>5.64	48.9±10.6		>6.28	-	
	**GALC**			**IDUA**			**GAA**		
	<1.40	60.0±10.7	**0.0295**	<4.94	53.2±13.4	0.368	<5.39	54.5±12.5	0.324
	1.40-1.86	44.4±14.3		4.94-7.08	48.9±11.1		5.39-7.93	50.6±9.7	
	1.86-2.54	52.8±12.9		7.08-10.11	49.6±8.39		7.93-10.18	45.7±4.9	
	>2.54	49.4±11.1		>10.11	58.2±15.4		>10.18	49.3±14.2	
	Substrate concentrations								
	**HexSph**			**LysoGb3**			**LysoSM**		
	<1.75	57	0.957	<0.54	45	0.554	<3.22	43±2.65	0.153
	1.75-2.97	48.4±10.1		0.54-0.78	59±13		3.22-3.96	48.5±12.8	
	2.97-4.61	52.4±11.5		0.78-1.89	51.5±12.0		3.96-4.87	52.1±10.8	
	>4.61	51.2±11.9		>1.89	50.4±11.3		>4.87	52.7±13.5	
	**Alpha-synuclein level**								
	<3.63	50.8±10.3	0.627						
	3.63-6.54	50.4±9.83							
	6.54-10.54	50.0±13.5							
	>10.54	52.7±12.7							

Table S3. List of selected rare deleterious variants of LSDs genes in SCZ patients.

Gene symbol	ID Pa-tients	rsID	Chr	Location	Transcript	Exon	Variant	Amino acid change	ExAC_ALL
HGSNAT	7	rs766835582	chr8	43037306	NM_152419	exon 11	c.G1031A	p.R344H	1.656e-05
ARSA	4	rs201251634	chr22	51065287	NM_001085427	exon4	c.C659T	p.P220L	0.0024

IDUA	5	rs532731688	chr4	996675	NM_000203	Exon9	c.C1345A	p.H449N	0.0008
IDUA	10	rs532731688	chr4	996675	NM_000203	Exon9	c.C1345A	p.H449N	0.0008
IDUA	14	rs74385837	chr4	995586	NM_000203	Exon3	c.C709T	p.L237F	0.0023

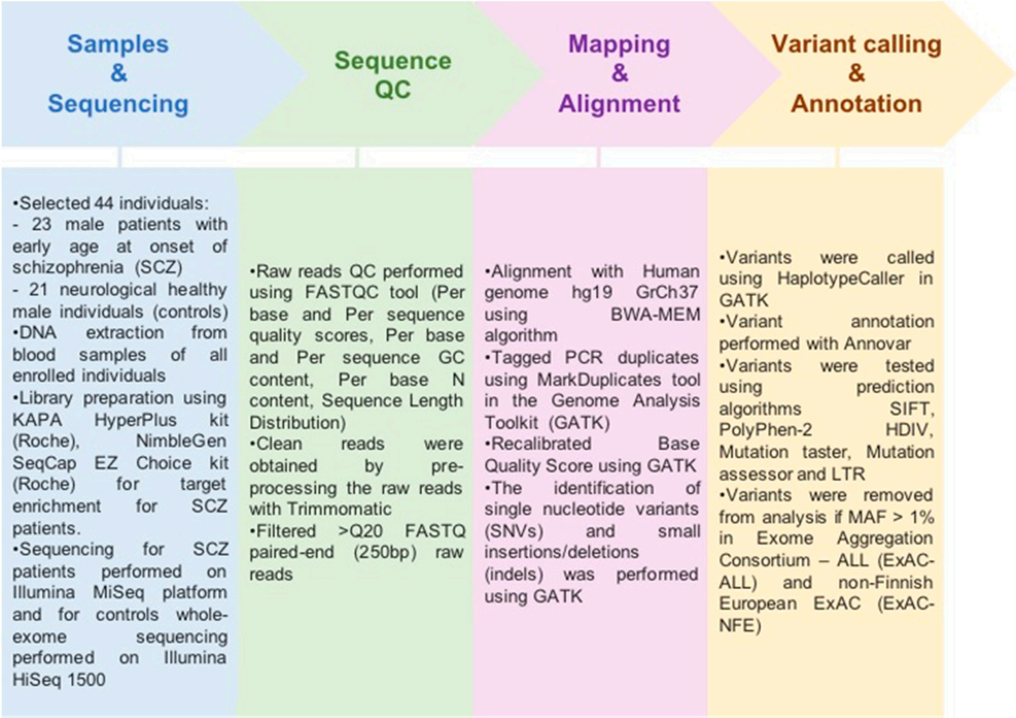

Figure S1. Block diagram of pipeline with numbers indicating a reduction in the total number of variants in each prioritization step.

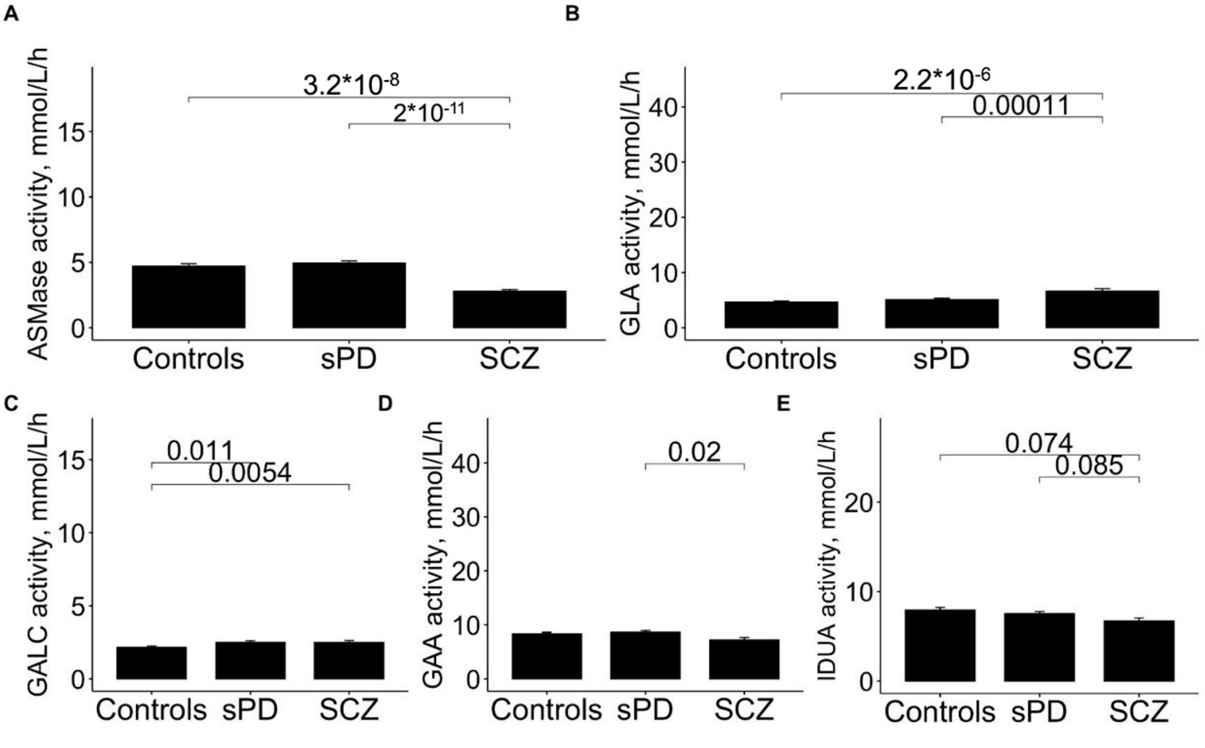

Figure S2. Activity of lysosomal enzyme in blood of studied groups. A. ASMase, B. GLA, C. GALC, D. GAA, E. IDUA.

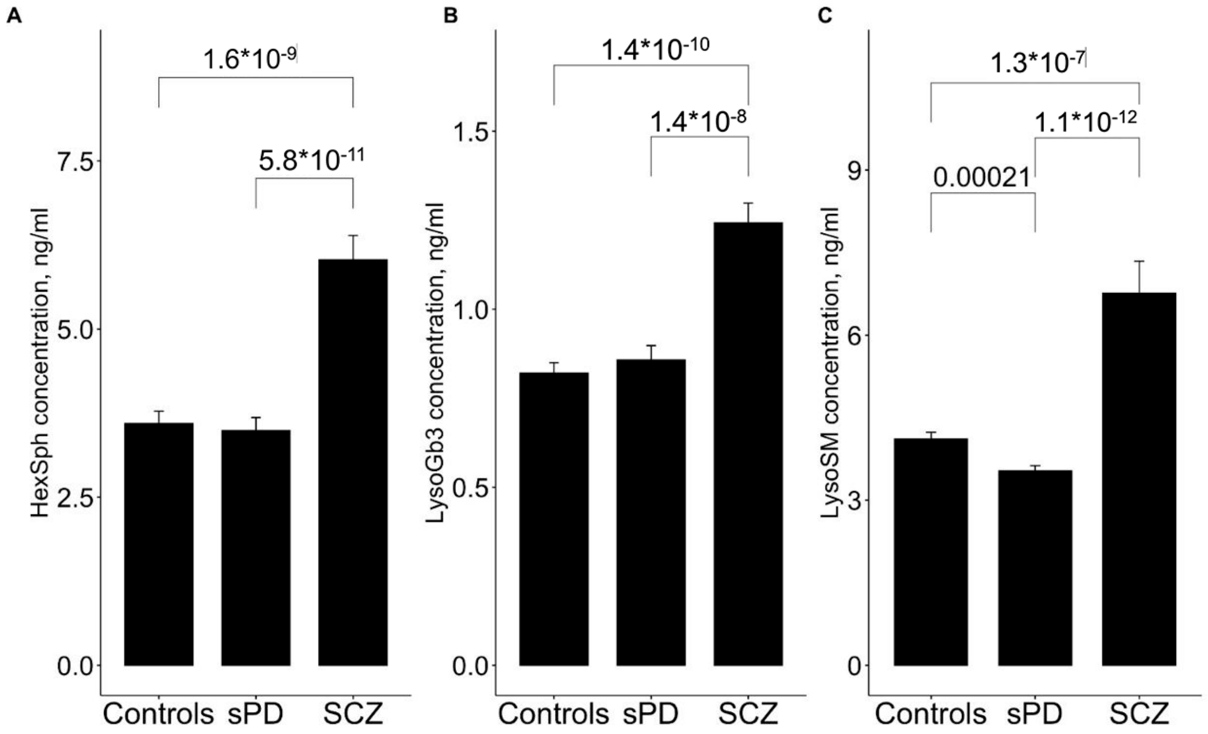

Figure S3. Substrate concentrations in blood and alpha-synuclein level in CD45+ blood cells of studied groups: A. HexSph, B. LysoGb3, C. LysoSM.

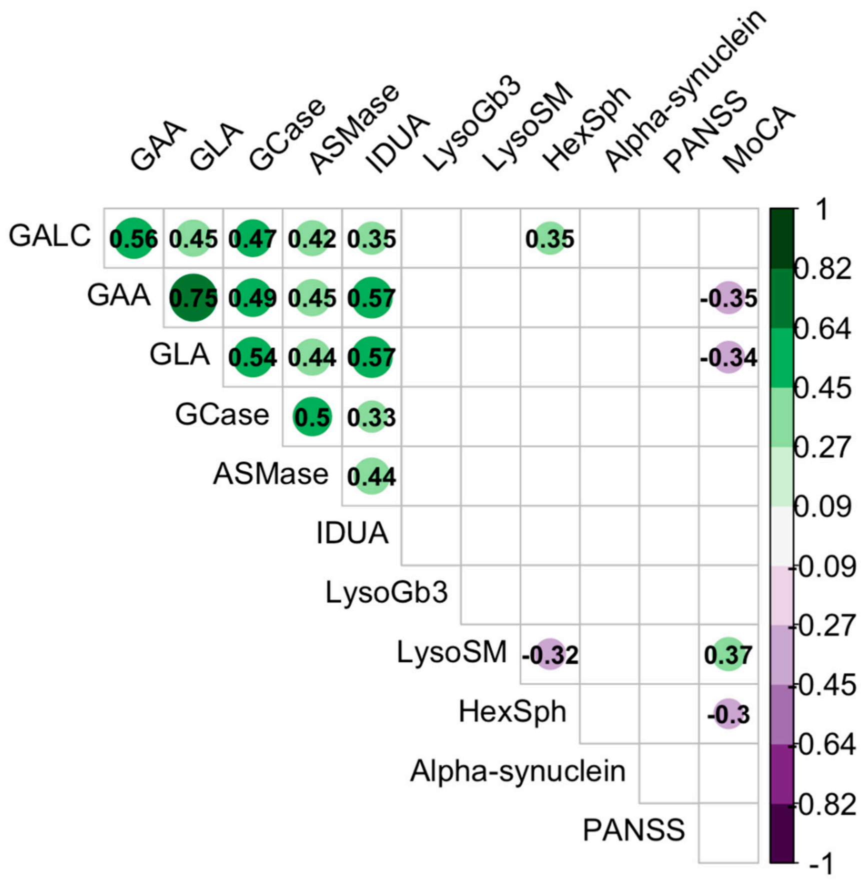

Figure S4. Correlation matrix among enzymes activities, substrate concentrations, and alpha-synuclein level in patients with late-onset SCZ. The violet and green dots correspond to negative and positive correlations, correspondently. Small dots with light colors represent lower correlations. Larger dots with darker colors correspond to higher correlations. White square—no statistically significant association, $p > 0.05$).
